# Multimodal ultrasound imaging of primary thyroid schwannoma: A case report

**DOI:** 10.1097/MD.0000000000025517

**Published:** 2021-04-23

**Authors:** Hai Na Zhao, Bu Yun Ma, Feng Yan, Yu Lan Peng

**Affiliations:** Department of Ultrasound, West China Hospital of Sichuan University, Chengdu, China.

**Keywords:** contrast-enhanced ultrasonography, elastography, guided biopsy, schwannoma, thyroid

## Abstract

**Rationale:**

Primary schwannoma of the thyroid gland is very rare, and its preoperative diagnosis is difficult.

**Patient concerns:**

We report the case of a thyroid nodule in an 18-year-old woman, who presented with a mass in her left neck with stiffness and normal thyroid function. However, the patient complained of numbness in her left upper extremity, and ultrasound (US) features were suspicious of malignancy.

**Diagnoses:**

Multimodal US imaging was performed using B-mode, color doppler, ultrasonic elastography (UE), and contrast-enhanced ultrasound (CEUS). CEUS revealed heterogeneous enhancement and “target sign” within the tumor. The nodule was suspicious for malignancy and classified as TI-RADS 4b, while the elasticity values measured by UE indicated a benign lesion. Fine needle aspiration (FNA) was subsequently performed in the markedly contrast-enhanced area for biopsy. Cytological results revealed a benign schwannoma.

**Intervention:**

The patient underwent left lobe resection. Postoperative pathology confirmed it to be a primary benign schwannoma of the thyroid.

**Outcomes:**

After thyroidectomy, the patient was followed-up with US. At present, all laboratory tests and thyroid imaging are normal, and the numbness of the left upper limb has disappeared.

**Lessons:**

The combination of different US modalities is useful for the diagnosis of thyroid lesions. FNA performed under CEUS guidance improves the accuracy of biopsy sampling.

## 1 Introduction

Schwannomas are benign tumors that arise from the Schwann cells of the nerve roots. They mainly arise from cutaneous or peripheral nerves, particularly in the neck, which accounts for approximately 25% of the cases. Schwannomas can occur at any age, but most patients develop the disease between the ages of 40 and 60 years.^[1]^

Primary thyroid schwannomas are very rare, and most previously reported cases were not definitively diagnosed prior to surgery, even with the fine-needle aspiration (FNA) strategy. Here, we report a case of primary thyroid schwannoma that was correctly diagnosed following FNA.

## 2 Case presentation

**Figure 1:**
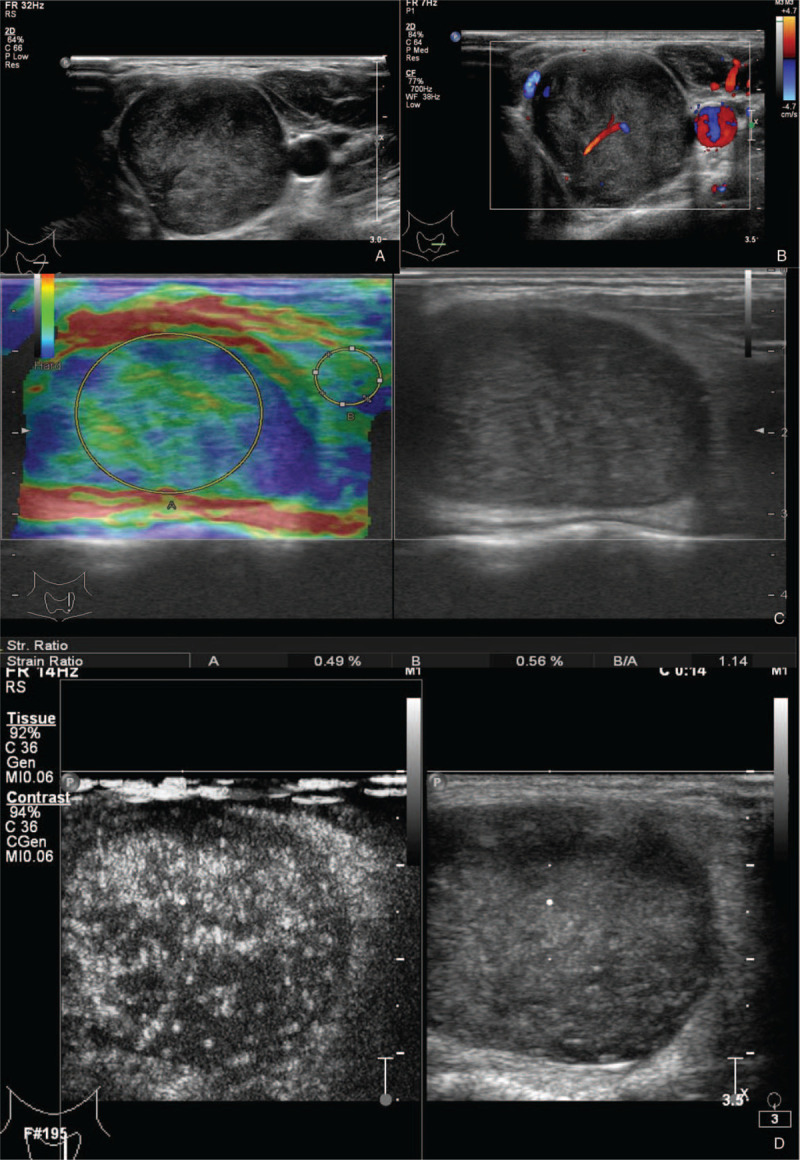
Multimodal ultrasound (US) imaging performed in a patient with a thyroid nodule suspicious for malignancy. The nodule size was 3.3 cm × 2.7 cm × 2.6 cm. (A) B-mode US revealed that the nodule was heterogeneous, hypo/iso-echoic with a sharp margin. (B) Color doppler showed intramural blood flow within the nodule with Adler grade I. (C) Ultrasonic elastography indicated that the lesion was medium-hard with a strain ratio of 1.14. (D) Contrast-enhanced ultrasound (CEUS) showed non-homogeneous enhancement (left) with “target sign” at baseline (right). B-mode and Color doppler were performed using the Philips IU 22 with a flat base 5 to 12 MHz probe and CEUS with a flat base 3 to 9 MHz probe. Ultrasonic elastography was performed using a HITACHI Vision 900 system.

**Figure 2:**
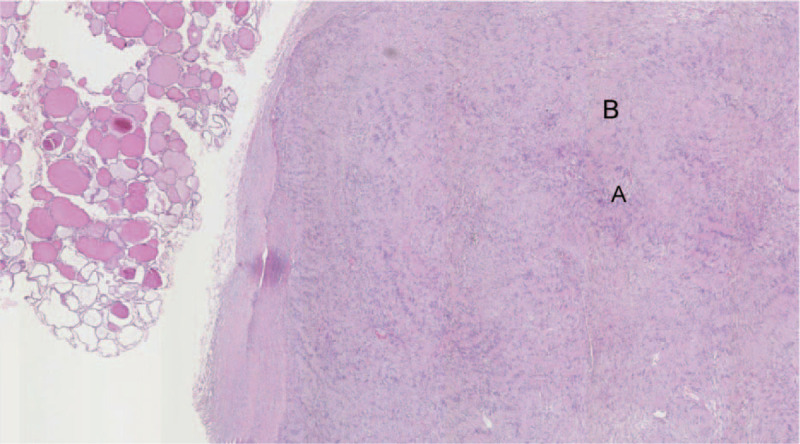
Micrograph of schwannoma after thyroidectomy (hematoxylin eosin staining; magnification, ×40). Antoni A areas, marked “A” on the image, are richer in tumor cells with several microvessels compared to Antoni B areas, marked “B,” which are characterized by the absence of blood vessels.

An 18-year-old woman presented to our hospital with a thyroid nodule. As the nodule was suspected to be malignant, and the patient complained of numbness in her left upper extremity, multimodal ultrasound (US) imaging was performed using B-mode, color doppler, ultrasonic elastography (UE) and contrast-enhanced ultrasound (CEUS). The nodule measured 3.3 cm in diameter, and demonstrated a sharp margin, heterogeneous echogenicity, and “target sign” in B-mode US (Fig. 1A), and intramural blood flow on color Doppler (Fig. 1B). As the nodule was suspected to be malignant, further examination was performed using UE and CEUS. UE showed that the thyroid nodule was of medium hardness with a strain ratio of 1.14 (Fig. 1C), indicating that the lesion was benign. CEUS was performed by injecting 1 ml of the contrast agent Sonovue (Bracco, Italy). The results revealed that malignancy could not be excluded, and the lesion was classified as TI-RADS 4b (Fig. 1D). Fine needle aspiration (FNA) (22-G) was performed under CEUS guidance and markedly enhanced areas were targeted for biopsy. FNA cytological results revealed that the lesion was a benign schwannoma. A partial thyroidectomy was performed. Intraoperatively, the tumor was found to be an encapsulated mass over the left lobe of the thyroid gland, without involvement of the surrounding structures. Hematoxylin and eosin (H-E) stained sections revealed that the resected tissue was composed of fusiform spindle-shaped cells densely arranged in fascicules (Fig. 2). The final diagnosis was a benign primary thyroid schwannoma. After thyroidectomy, the patient was followed up by US for 3 years. All laboratory tests and thyroid imaging were found to be normal, and the numbness of the left upper limb disappeared.

### 2.1 Ethical review

This was a retrospective review of medical records, and the requirement for ethical approval and informed consent was waived.

## 3 Discussion

Schwannomas are peripheral nerve tumors that originate from the neuronal sheath cells. Like other non-epithelial tumors found in the thyroid gland, schwannomas are extremely rare. To our knowledge, only 22 cases have been reported in the English literature. The age of patients with schwannomas ranged from 12 to 77 years.

Primary thyroid schwannomas originate from intrathyroidal sensory nerves, or sympathetic and parasympathetic innervations.^[4]^ Most thyroid schwannomas are characterized by an oval shape, well-defined margin, along with hypoechoic, homogenous, and poorly vascularized nodules on conventional US.^[1–8]^ However, these characteristics overlap with other common thyroid nodules, such as goiters and adenomas. Thus, it is difficult to make a differential diagnosis. Unlike schwannomas originating from the peripheral nerves, primary thyroid schwannomas demonstrate no typical “rat tail sign” on US. According to the English literature, there are only 8 cases of thyroid schwannomas detected by US.^[1–8]^ The imaging findings are summarized in Table 1. In a recent study, Yang et al^[9]^ reported that 44.4% of schwannomas of the peripheral nerves showed a “target sign” on US images, which is also one of the characteristic features of schwannomas on magnetic resonance imaging (MRI). Through careful examination of the previously reported US images,^[1–8]^ we found that one-third of thyroid schwannomas also demonstrated the “target sign,” consistent with this case (Fig. 1A and 1D). Therefore, the “target sign” may be a distinctive US feature of thyroid schwannomas, which can distinguish them from other common thyroid nodules.

**Table 1:** Summary of recent cases of thyroid schwannoma with ultrasonic imaging including.

Reference	US features	Primary US diagnosis	Preoperative FNA finding
Dhar H^[1]^	A well defined, hyperechoic solid lesion with cystic degeneration in the right lobe, without target sign	None	A benign lesion
Chen G^[2]^	A well defined hypoechoic solid lesion in the left lobe, without target sign	None	None
Jong YN^[3]^	A polygonal hypoechoic lesion without calcification in the left lobe, target sign	None	Inconclusive
Aoki T^[4]^	A well defined, mostly solid lump without calcification in the right lobe, without target sign	A benign tumor	None
Giuseppa GCC^[5]^	A well defined hypoechoic solid lesion in the isthmus, without target sign	Nodular goiter	Nodular goiter
Young SL^[6]^	A lobulated, heterogeneous and hypoechoic nodule in the right lobe, target sign	None	A paucicellular smear composed of bland spindle-shaped cells in loosely cohesive aggregates.
Shaher Abbarah^[7]^	A well defined, large heterogeneous, predominantly cystic nodule in the left lobe, without target sign	None	Atypia of undetermined significance
GVázquez-Benítez^[8]^	A well defined hypoechoic solid lesion in the left lobe, without target sign	None	Benign mesenchymal cells
The present case	A well defined hypoechoic solid lesion in the left lobe, target sign	Suspicious malignancy	A benign schwannoma

UE is a new, noninvasive, and cost-effective diagnostic tool that can improve the accuracy of assessing the malignancy risk of solid thyroid nodules.^[10]^ In many hospital centers, elastography is integrated into routine US examination of thyroid nodules. In this study, we also attempted UE to differentiate between benign and malignant nodules. The examined nodule was of medium hardness with a strain ratio of 1.14 (Fig. 1C), which was significantly lower than that of malignant nodules, indicating that the lesion in this case was benign.^[10,11]^ Thus, UE could be useful in distinguishing schwannomas from other common thyroid cancers.

To our knowledge, no contrast-enhanced ultrasound study has been performed on thyroid schwannomas. Only 3 cases of peripheral nerves schwannomas were studied with CEUS, and 1 with combined CEUS and UE.^[12–14]^ These 3 schwannomas were located in the liver, pancreas, and the common fibular nerve. Both hepatic and pancreatic nodules contained multiple cysts with mixed internal septations and solid areas on CEUS, similar to the MRI findings.^[13,14]^ In our case, the tumor presented heterogeneous enhancement without liquefaction, which was a significant difference from the above cases. According to the CEUS examination in our case, the nodule was classified as TI-RADS 4b, which contradicted the result indicated by elastography.

According to our experience, even with the strategy of FNA biopsy, it is difficult to obtain a correct preoperative diagnosis of thyroid schwannoma. The cause of poor diagnosis or misdiagnosis is probably incorrect sampling.^[17]^ Ultrasound-guided FNA may provide better precision, and CEUS may help to detect the suspicious lesion as well as to delimit the avascular necrotic areas from the viable vascularized regions of the thyroid. Successful retrieval of an adequate tissue sample is achievable in only 80% to 90% of the cases, mainly due to lack of tissue homogeneity in the lesions.^[16,17]^ CEUS has been demonstrated to improve the success of percutaneous biopsy in several tumors.^[16,17]^ In the present study, when FNA was performed, the needle was placed directly into the markedly contrast-enhanced areas, which allowed for the retrieval of viable tumor cells, thus yielding a high degree of specimen viability. Pathologically, schwannomas are composed of Schwann cells with regions of dense high-cellular components in the Antoni A areas mixed with loose myxoid degenerative Antoni B areas. Antoni A areas are richer in microvessels than Antoni B areas,^[10]^ and are more hyperenhanced. Therefore, CEUS enables the delimitation of Antoni A from the Antoni B areas of the tumor for FNA guidance.

Generally, our policy of multimodal ultrasound imaging is as follows: when a lesion is suspected on conventional US, a second imaging modality will be applied, such as UE; if there is discordance between the 2 methods, CEUS-guided biopsy will be performed, particularly for larger nodules.

In conclusion, we successfully diagnosed a thyroid schwannoma prior to surgery using CEUS-guided fine-needle aspiration. Multimodal ultrasound (B-mode, color Doppler, UE, and CEUS) is useful for diagnosing thyroid schwannoma and differentiating it from other thyroid nodules. UE is helpful for the differentiation of benign and malignant lesions, while CEUS is very useful for improving the accuracy of FNA biopsy and the success rate of thyroid surgery.

## Acknowledgments

We are thankful to Xiang-Lan Zhu from the department of pathology, West China hospital of Sichuan University, who provided the histopathological diagnosis of the case.

## Author contributions

**Conceptualization:** Hai Na Zhao, Feng Yan, Yu Lan Peng.

**Data curation:** Hai Na Zhao, Bu Yun Ma.

**Formal analysis:** Hai Na Zhao, Feng Yan, Yu Lan Peng.

**Supervision:** Feng Yan, Yu Lan Peng.

**Writing – original draft:** Hai Na Zhao.

**Writing – review & editing:** Yu Lan Peng.

## Correction

The corresponding author for the article has been changed from Dr. Feng Yan to Dr. Yu Lan Peng. The corresponding author information has been updated accordingly.
